# Reference exome data for a Northern Brazilian population

**DOI:** 10.1038/s41597-020-00703-y

**Published:** 2020-10-21

**Authors:** Alexia L. Weeks, Richard W. Francis, Joao I. C. F. Neri, Nathaly M. C. Costa, Nivea M. R. Arrais, Timo Lassmann, Jenefer M. Blackwell, Selma M. B. Jeronimo

**Affiliations:** 1 Telethon Kids Institute, The University of Western Australia, Perth Children’s Hospital, Western Australia Perth, Australia; 2 Institute of Tropical Medicine of Rio Grande do Norte and Department of Biochemistry, Universidade Federal do Rio Grande do Norte, Natal, Rio de Grande do Norte, Natal, Brazil; 3 Department of Pediatrics, Federal University of Rio Grande do Norte and Empresa Brasileira de Serviços Hospitalares, Natal, Brazil; 4 National Institute of Science and Technology of Tropical Diseases, Natal, RN Brazil

**Keywords:** Genetics research, Medical genetics

## Abstract

Exome sequencing is widely used in the diagnosis of rare genetic diseases and provides useful variant data for analysis of complex diseases. There is not always adequate population-specific reference data to assist in assigning a diagnostic variant to a specific clinical condition. Here we provide a catalogue of variants called after sequencing the exomes of 45 babies from Rio Grande do Nord in Brazil. Sequence data were processed using an ‘intersect-then-combine’ (ITC) approach, using GATK and SAMtools to call variants. A total of 612,761 variants were identified in at least one individual in this Brazilian Cohort, including 559,448 single nucleotide variants (SNVs) and 53,313 insertion/deletions. Of these, 58,111 overlapped with nonsynonymous (nsSNVs) or splice site (ssSNVs) SNVs in dbNSFP. As an aid to clinical diagnosis of rare diseases, we used the American College of Medicine Genetics and Genomics (ACMG) guidelines to assign pathogenic/likely pathogenic status to 185 (0.32%) of the 58,111 nsSNVs and ssSNVs. Our data set provides a useful reference point for diagnosis of rare diseases in Brazil. (169 words).

## Abstract (summary)

**Table:** 

Measurement(s)	exome • Exon Mutation
Technology Type(s)	DNA sequencing assay • comparison analysis operation
Sample Characteristic - Organism	Homo sapiens
Sample Characteristic - Location	Rio Grande do Norte State

Machine-accessible metadata file describing the reported data: 10.6084/m9.figshare.12839735

## Background & Summary

Next-generation sequencing of protein-coding regions, known as whole exome sequencing (WES), has enabled molecular diagnoses for thousands of rare disease patients (reviewed^1^) and provides useful variant data for genetic studies of complex diseases. As the use of this technology spreads world-wide it is becoming more important to understand genetic heterogeneity at a population-specific level, and to generate adequate population-specific reference data to assist clinical geneticists in assigning a diagnostic variant to a specific clinical condition. One region in which this is becoming increasingly important is in South America, and more specifically in Brazil, where genetic causes of clinical traits such as congenital microcephaly, ocular disease, need to be differentially diagnosed from those associated with Zika virus infection acquired *in utero*. In examining a cohort of 45 Brazilian babies from the State of Rio Grande do Norte we undertook WES to ascertain that none of 44 babies presenting with Zika-associated microcephaly were due to pathogenic genetic variants known to be associated with this clinical trait. One baby presented with familial congenital microcephaly. Here we describe the baseline data on variants identified in this population, with a specific focus on known and novel (i.e. those exclusive to this Brazilian population) rare variants that will inform the diagnosis of rare genetic diseases in Brazil. The data also provides useful information on novel and common variants that add to our knowledge of genetic heterogeneity in Brazil and may contribute to studies of genetic risk factors for complex diseases.

**Fig. 1:**
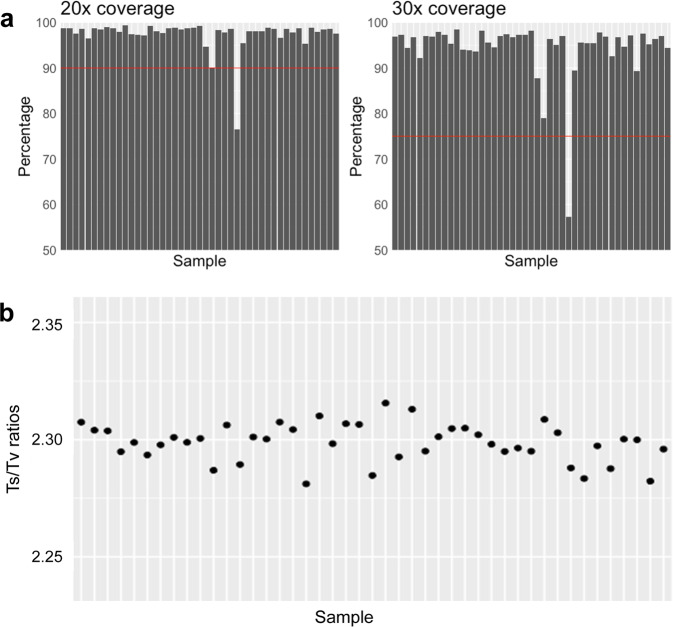
(**a**) WES coverage for the at 20X and 30X depth. Each bar represents an individual sample and the percentage of bases with at least 20X or 30X coverage. The red lines mark the 90% and 75% coverages at 20X and 30X depths, respectively, which are optimal targets for WES that most of the samples achieved. (**b**) Ts/Tv ratio calculated individually for all individuals using SNVs passing the GATK best practice VQSR threshold.

The exome data were processed with GATK 4.0.2.0^2,3^ and SAMtools 1.7^4^ using an ‘intersect-then-combine’ (ITC) approach. Variant calling was performed using the GATK best practices and SAMtools mpileup, only variants identified by both methods were retained. We calculated an average sequence depth of 97.4% at 20X coverage and 94.4% at 30X coverage (Fig. 1a). An average transition/transversion (Ts/Tv) ratio of 2.30 was observed for the Brazilian sample used here (Fig. 1b).

**Fig. 2:**
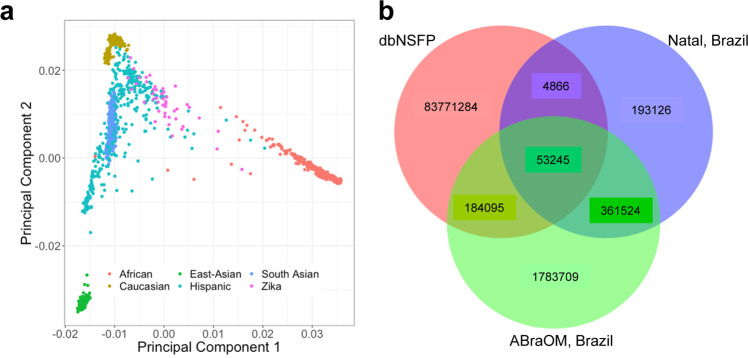
(**a**) PCA plot to demonstrate ethnic admixture in the Brazilian sample compared to 1000 G populations grouped as African (ACB, ASW, ESN, GWD, LWK, MSL, YRI); Hispanic (CLM, MXL, PEL, PUR); East-Asian (CDX, CHB, CHS, JPT, KHV); South Asian (BEB, GIH, ITU, PJL, STU); and Caucasian (CEU, FIN, GBR, IBS, TSI). (**b**) Venn diagram showing overlap between SNVs in two Brazilian samples (ABraOM database of exome variants from 609 elderly Brazilians from Sao Paulo State^5^ and the 45 exomes from Rio de Grande do Norte State studied here) compared to large public domain databases.

Sequences were aligned to the hg19 reference human genome and a total number of 612,761 variants were identified in at least one individual. Of these variants, 559,448 were single nucleotide variants (SNVs) and 53,313 were insertions/deletions (indels). To evaluate admixture in this Brazilian sample we carried out principal component analysis on an LD-pruned set of SNVs with minor allele frequencies >0.1. Comparison with 1000 G populations indicated predominant admixture between Caucasian and Negroid populations (Fig. 2a), consistent with data from the ABraOM database of exome variants from 609 elderly Brazilians from Sao Paulo State^5^. In comparing our data with the ABraOM database we found 414,769 variants in common with the ABraOM study and 197,992 that were unique to our study sample. Comparing the data with large public domain datasets (dbSNP 151^6^, 1000 Genomes Phase 3^7^, TWINSUK^8^, ESP6500^9^, UK10K^10^, ExAC^11^ and gnomAD^12^ databases) we found 361,524 variants that were unique to the combined Brazilian datasets (Fig. 2b).

**Fig. 3:**
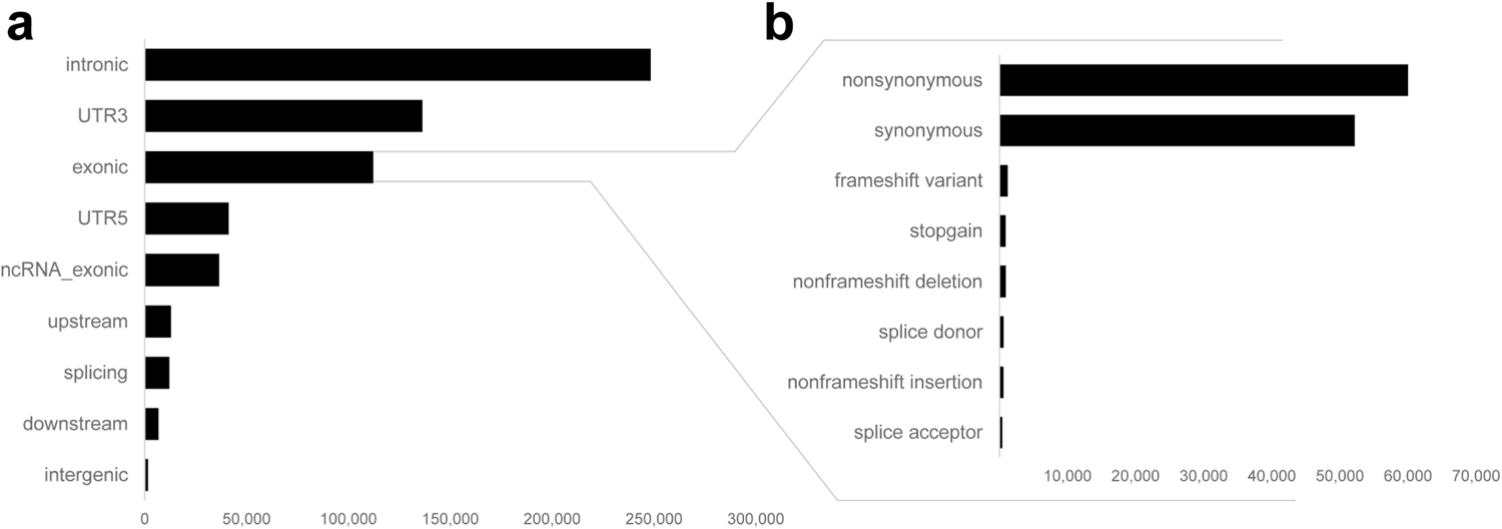
Annotation of identified variants reported by (**a**) genomic location and (**b**) the main variant consequences. Other variant consequences not in the figure included: stop_lost (164); start_lost (219); protein_altering_variant (4); incomplete_terminal_codon_variant (8); stop_retained_variant (72); coding_sequence_variant (16); and mature_miRNA_variant (160).

The 612,761 variants in our study sample were annotated with VEP^13^ to provide variant types and consequences (Fig. 3). Most variants were categorised as intronic, exonic or UTR3, consistent with design of the exome sequencing capture kit. A total of 248,329 intronic variants, 117,524 exonic variants and 136,266 UTR3 variants were present.

Exome variants of interest in diagnosis of rare genetic diseases usually fall within the categories of nonsynonymous SNVs (nsSNVs) and splice‐site variants (ssSNVs). To identify this potentially functional subset of variants in our dataset we looked for overlap between our variants and those present in the dbNSFP v4.0^14,15^ database of human nsSNVs and ssSNVs. Using the search_dbNSFP40a function we identified 58,111 nsSNVs/ssSNVs in our sample that were present in dbNSFP.

To further identify nsSNVs/ssSNVs that may be pathogenic for genetic diseases we determined the number that classify as “pathogenic” or “likely pathogenic” according to the American College of Medicine Genetics and Genomics (ACMG) standards and guidelines^16^ (Supplementary Table 1). Of the 58,111 nsSNVs/ssSNVs in our sample a total of 12 (0.02%) were classified as “pathogenic” and 173 (0.30%) as “likely pathogenic”. Details of these variants is provided in Supplementary Table 2.

## Methods

### Study population

Subjects were recruited through the Pediatric Hospital of the Federal University of Rio Grande do Norte or through visits to households that had cases suspected of microcephaly in Natal and other cities where cases of microcephaly were reported during the 2015–2016 ZIKV outbreak, Rio Grande do Norte, Brazil. The sample comprised 45 babies (26 males aged mean ± SD 25.50 ± 7.17 months; 19 females aged mean ± SD 24.79 ± 6.73 months), 44 with confirmed Zika-associated congenital microcephaly and one baby with familial congenital microcephaly. None of the babies with Zika-related microcephaly had a deleterious genetic variant previously known to be associated with genetically determined congenital microcephaly that could account for their phenotype (see Supplementary Table 2). Nor did we find a variant that matched deleterious variants in dbNSFP v4.0 that would account for the one familial case of microcephaly. The complete list of genes and filtering strategy that we applied to look for microcephaly variants is provided in Supplementary Table 3.

### Ethical considerations

This study was undertaken with ethical approval from the institutional review board of the Universidade Federal do Rio Grande do Norte/Comissão Nacional de Ética em Pesquisa (CAAE 53111416.7.0000.5537). Written consent was obtained from the parents or legal guardians of babies who ranged in age from 5 months to 40 months. The individual consent included an option to accept or refuse continued use of their genetic or clinical data in further studies. The parents or legal guardian of all subjects included in the study had given consent for storage and future use of deidentified DNA samples and data for their children.

### Whole exome sequencing

The DNA samples were prepared following the Agilent SureSelect XT + UTR v6 protocol and sequenced on a HiSeq. 4000 system using 150 bp paired end chemistry at the Genomics Division, Iowa Institute of Human Genetics, University of Iowa, USA. Sequence data was processed with GATK 4.0.2.0^2,3^ and SAMtools 1.7^4^ using an ‘intersect-then-combine’ (ITC) approach. Variant calling was performed with GATK following best practices^17^ and with SAMtools^4^ using the mpileup function. Only variants identified by both methods were retained. Sequence coverage was calculated using BEDtools^18^ with the -d parameter to calculate the per base depth and then the percentage of bases with at least 20X and 30X coverage were calculated.

### Variant annotation

Prior to annotation, the data were normalized and decomposed with VT v0.57721^19^. Variant annotation was performed using the Variant Effect Predictor (VEP v97.3)^13^. VEP annotated variants as splicing, ncRNA, UTR5, UTR3, intronic, upstream, downstream or intergenic, with exonic variants categorised as start lost, stop lost, stop gain, frameshift insertion, frameshift deletion, nonframeshift insertion, nonframeshift deletion, protein altering variant, incomplete terminal codon variant, stop retained variant, coding sequence variant, mature miRNA variant, missense SNV or synonymous SNV.

### Overlap with known variants

Variants in our dataset were compared with data from the Brazilian ABraOM databases^5^ and with large public domain datasets dbSNP 151^6^, 1000 Genomes Phase 3^7^, TWINSUK^8^, ESP6500^9^, UK10K^10^, ExAC^11^ and gnomAD^12^ databases. To gain a handle on functionality relevant to rare disease diagnosis, variants in our sample were also compared to the 84,013,490 nsSNVs and ssSNVs present in the dbNSFP^14,15^ v4.0 database.

### Principal component analysis

Scripts used to perform the data processing and plotting for principal component analysis are available at https://github.com/richardwfrancis/zika_admixture.

### Classification of variant pathogenicity

To further identify nsSNVs/ssSNVs that may be pathogenic for genetic diseases we determined whether they classified as “pathogenic” or “likely pathogenic” according to the ACMG standards and guidelines^16^ using criteria laid out in Supplementary Table 1 and implemented using the python script available here: https://github.com/TimoLassmann/Phenoparser/blob/master/scripts/acmg.py.

## Data Records

The full set of variants has been recorded as two VCF files: (i) The complete normalised and VEP annotated version containing only PASSed variants and (ii) the subset of variants found in dbNSFP, which is further annotated with ACMG classification. These files have been deposited in the European GenomePhenome Archive (EGA) under the accession number EGAS00001004112^20^. Summary allele frequency data have also been deposited in the European Nucleotide Archive (ENA) under the project and analysis accession numbers PRJEB39409 and ERZ1466912, respectively^21^.

## Technical Validation

The Ts/Tv ratio was calculated for each sample using the stats function in BCFtools as a quality control metric. It has been reported that a Ts/Tv ratio of 2.8 is expected for WES^22^. However this varies greatly by genome region and functionality^23^, and is usually ~3.0 for exome regions and ~2.0 outside of exome regions^24^.

## Usage Notes

Summary allele frequency data are available as open access through the ENA. The variant data are made available through the EGA. Access to variant data will be granted to qualified researchers for appropriate health related uses, subject to review by a study-specific Data Access Committee (DAC).

## Supplementary information

Supplementary Table 1

Supplementary Table 2

Supplementary Table 3
