# Supplementary material for: Reference exome data for a Northern Brazilian population
Source: Sci Data. 2020 Oct 21;7:360. doi: 10.1038/s41597-020-00703-y (PMC7578642; doi:10.1038/s41597-020-00703-y)
Supplement: Supplementary file 1 — Supplementary Table 1 [file 41597_2020_703_MOESM1_ESM.docx]

**Supplementary Table 1** Criteria used to assign pathogenicity criteria to variants following ACMG guidelines. To accompany some of the methods (pvs1, pp2) we created a gene:impact:disease database (GIDdb) compiled using data from Ensembl and OMIM that links HGNC gene symbols to OMIM diseases via the sequence ontology impact of the known causal variant.

| Code | Criteria for code assignment |
| --- | --- |
| pvs1 | null variant in a gene where Loss of Function (LOF) is a known mechanism of disease |
| ps1 | Same amino acid change as a previously established pathogenic variant regardless of nucleotide change |
| pm1 | Located in a mutational hot spot and/or critical and well-established functional domain without benign variation |
| pm2 | Absent from controls (or at extremely low frequency if recessive) |
| pm4 | Protein length changes as a result of in-frame deletions/insertions in a nonrepeat region or stop-loss variants |
| pm5 | Novel missense change at an amino acid residue where a different missense change determined to be pathogenic has been seen before |
| pp2 | Missense variant in a gene that has a low rate of benign missense variation and in which missense variants are a common mechanism of disease |
| pp3 | Multiple lines of computational evidence support a deleterious effect on the gene or gene product |
